# 2D materials coated on etched optical fibers as humidity sensor

**DOI:** 10.1038/s41598-020-79563-w

**Published:** 2021-01-19

**Authors:** Erfan Owji, Hossein Mokhtari, Fatemeh Ostovari, Behnam Darazereshki, Nazanin Shakiba

**Affiliations:** Factually of Science, Department of Physics, Yazd University, Yazd, Iran

**Keywords:** Nanoscience and technology, Optics and photonics

## Abstract

In this investigation, etched-fibers are coated by 2D layers such as Molybdenum disulfide (MoS_2_), Molybdenum diselenide (MoSe_2_) and composition of graphene and graphene oxide (G/GO) to modify humidity sensing. The relative differentiation of attenuations (RDA) in presence of relative humidity (RH) is measured by Optical Loss Test Set at two standard-wavelengths-telecommunication (1310 nm and 1550 nm). Results show that the etched single-mode fiber (ESMF) coated with G/GO has relatively high and one by one function for RDA versus RH (more than 30%). Also, its sensitivity and variance are reasonable. The MoSe_2_ based sensor is applicable at humidity below 30% because of higher RDA. However, it is not useful at humidity more than 30% due to the absence of one by one function for RDA versus RH. Besides, ESMF coated with MoS_2_ has indistinctive behavior and is not useful as a humidity sensor.

## Introduction

Sensors based on optical fibers have a key role in industrial, biomedical, and home security purposes. The great advantages of optical sensors such as high speed, insensitive to electromagnetic interference, anti-explosion, small size, low weight, durable and chemically inert, and low transmission make them practical and affordable^1,2^. However, optical fiber sensors can be used to measuring different physical properties, such as measurements of RI, strain^3^, temperature^4,5^, and humidity^4–10^. The mechanism of these sensors is very dependent on changes in the refractive index^3,4^. Usually, these sensors are made by modifying the geometry of optical fiber such as side-polished fiber, a long period fiber grating (LPG), and hollow-core fiber sensors that are very complicated^5–13^. Recently, in order to improve the sensing properties, coating of optical fibers by nanomaterials has been used.

The humidity sensor is one of the most widely used sensors. So far, extensive researches have been done on the optical humidity sensors due to their advantages. However, the very low permeability of the protective layer and clad of fiber to the vapor leads to slight changes in the refractive index and so very poor sensitivity and non-linearity response. Researchers used physical and chemical changes on the external surface of optical fiber to improve humidity sensing. Especially, coating of two dimensional (2D) materials have attracted great attention in high-sensitive sensors due to their high surface to volume ratio and other intrinsic properties^14–16^. In addition, the ability to absorb water molecules depends on the surface’s functional groups of 2D materials. Electrical gap and consequently refractive index changes with bonding between the water molecule and surface of coated optical fiber.

Graphene (G), as the first experimental 2D materials, has attracted a wide range of interest due to its fascinating electronic, mechanical, and thermal properties such as extremely high carrier mobility, mechanical flexibility, optical transparency, and chemical stability^17–23^. Among other applications, the exceptional surface to volume ratio and high electron mobility in room temperature entitles G as a promising candidate for sensing applications^24^. Usually, in the synthesis process, different functional groups create on the surface of G and change its properties. Oxygen functional groups such as hydroxyl, carboxyl, epoxide, and carbonyl are the main functional groups that convert graphene to graphene oxide (GO). Presence of these groups increases surface and interlayer adsorption of water molecules through the polar bonding^11,16,25^.

Different humidity sensors based on optical fiber with different geometrical shape coated with G or GO have been investigated. Although some groups have used humidity sensors based on fiber optic with reduced graphene coating due to changes in carrier density by variations of relative humidity^10,12,16,26,27^, others have preferred graphene oxide because of its hydrophilic property and changes in its refractive index by adsorption of water molecules between its layers^11,28–30^. It seems that by use of G and GO simultaneously, make it possible to use the benefit of both them in humidity sensing based on optical fiber which has not been studied until now.

Also, the layered transition metal dichalcogenides (TMD) materials—a new family of 2D materials have attracted huge attention due to tunable direct band gap, high mobility, low-level toxicity, and large surface area^15,31,32^. The monolayer of Molybdenum Disulfide (MoS_2_) and Molybdenum Diselenide (MoSe_2_) is an outstanding example, in which molybdenum atoms are sandwiched between two layers of Sulfur and Selenium atoms respectively. The humidity sensor based on the side-polished optical fiber coated with these nanosheets have been investigated at 1550 nm wavelength^33–35^. The use of mechanical polishing method results in insertion loss of the side-polished fiber and smoothness of edges is difficult.

However, chemical polishing methods have preserved the cylindrical shape of the optical fiber and is much simpler and more practical than other methods. As far as we have researched, so far the optical fibers that have been chemically thinned and layered with 2D materials have not been applied as humidity sensor.

In this study, for the first time, chemical etching the surface of single-mode optical fiber (SMF) by hydrofluoric acid (HF) as a simple polish method and coating 2D materials such as the composition of G and GO (G/GO) to use the advantage of both them, MoS_2_ and MoSe_2_ on the etched-SMF (ESMF) are used to investigate the humidity sensing properties of optical fiber. So, at first G/GO, MoS_2_ and MoSe_2_ are synthesized and qualified by Scanning Electron microscopy (SEM), Transmission Electron Microscopy (TEM), Energy-Dispersive X-ray spectroscopy (EDX), Raman, FTIR (Fourier-transform infrared spectroscopy) and XRD (X-ray Diffraction) analysis. Then, etched-SMFs are coated by synthesized 2D materials. In the end, the relative differentiation of attenuation (RDA), repeatability, sensitivity, and variance versus relative humidity (RH) at two standard- wavelengths- telecommunication (1310 nm and 1550 nm) are measured.

Also, the behavior of the introduced humidity sensor has been analyzed based on the surface functional groups and the semiconductor type (n-type or p-type) of the coated 2D materials on the ESMF. We have not found this type of analysis in the field of humidity sensing by fiber optic as far as we have studied.

## Materials and methods

In this research, SMF is used as the main part of the optical-humidity sensor. To modify the sensing, the diameter of SMF reduced by corroding it in HF acid and layered with 2D materials (G/GO, MoSe_2_, and MoS_2_) to achieve desirable evanescence field. The Optical Loss Test Set (OLTS) as an analyzer (Optical power source (OPS) and Optical power meter (OPM)) was used to measure RDA versus RH.

### Synthesis

#### Graphene

The G/GO layer is synthesized by Liquid Phase Exfoliation of Graphite Oxide. Usually, the composition of G and GO produces in this method. After synthesis of Graphite Oxide powder by Hummer’s method^36,37^, 5 mg of it disperses in 10 cc distilled water and sonicates to separate individual Graphite Oxide layers. The transparent liquid above sediments that contain few/mono-layer of G/GO is achieved after centrifugation at 6000 rpm for 3 h. The quality of G/GO layers is investigated by SEM, TEM, EDX, Raman, FTIR and XRD analysis.

**Figure 1:**
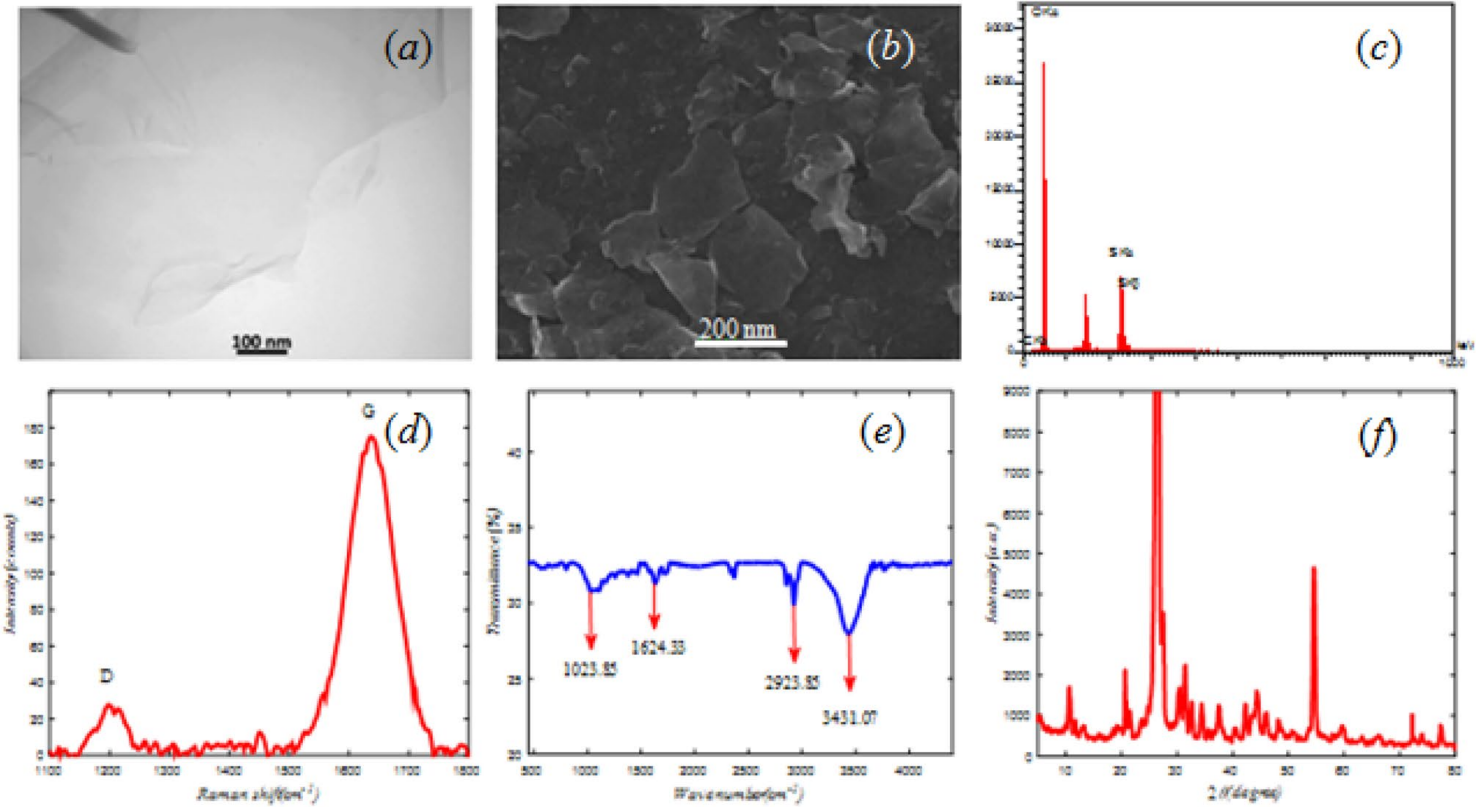
**(a)** TEM, **(b)** SEM, **(c)** EDX, **(d)** Raman, **(e)** FTIR, and **(f)** XRD analysis of G/GO.

The SEM and TEM images display the microstructure of synthesized G/GO (Fig. 1a,b). The corresponding EDX analysis (Fig. 1c) indicates the presence of some impurity in the results and high O/C ratio.

The Raman spectra of G/GO layer show two important peaks called D and G bands (D ~ 1197 and G ~ 1634 cm^−1^). The G and D bands correspond to the presence of sp^2^ carbon-type structures within the sample, and defects in the hexagonal structure of graphite respectively (Fig. 1d)^38,39^.

The presence of C–O, C=O and C–H bands at 1020, 1624 and 2,923 cm^−1^ respectively are evident from the FTIR spectra (Fig. 1e.). Also, the spread peak around 3430 cm^−1^ is due to the tensile vibration of phenol (C–OH) groups^40,41^.

The XRD of G/GO sheets (Fig. 1f) illustrates the peaks at 2θ = 10° (d001 = 0.83 nm), 2θ = 20° (d002 = 0 0.48 nm), 2θ = 26° (d002 = 0 34 nm) and 2θ = 55° (d004 = 0 0.17 nm). The peaks at 2θ = 26°, 55° is characteristic of graphite and after chemical oxidation of it, the inter planar distance increase and the other peaks appear^39,42^.

#### MoSe_2_

The synthesis of MoSe_2_ was down by Solvothermal method^24^ in normal ambient environmental conditions. In this method Se powder, Na_2_MoO_4_, distilled water, and Hydrazine Hydrate are mixed in a container. The materials are stirred constantly and heated with rate $2\;^\circ {\text{C/min}}$ for 45 min. Then, the temperature of the homogenize solution is maintained at 120 ℃ for an hour. Afterward, to increase the pH of the solution up to 12, Hydrazine Hydrate is gradually added and the color of the solution turned to dark brown.

After water evaporation by kept the solution in oven at 60 ℃ for 45 min, the residual powder was washed with water and ethanol 3 times and again was kept in oven at 60 ℃ for 10 h until all the water was evaporated and the dark powder was left.

To easy distribution of MoSe_2_ powder in water, the obtained powder is stirred in alcohol for 12 h, dried in the environmental conditions and finally, 5mgr of it dispersed in 10 cc distilled water and sonicates to separate individual layers.

The transparent liquid above sediments that contain few/mono-layer of MoSe_2_ is achieved after centrifugation at 6000 rpm for 3 h. The quality of MoSe_2_ layers is investigated by TEM, SEM, EDX, Raman, FTIR and XRD analysis.

**Figure 2:**
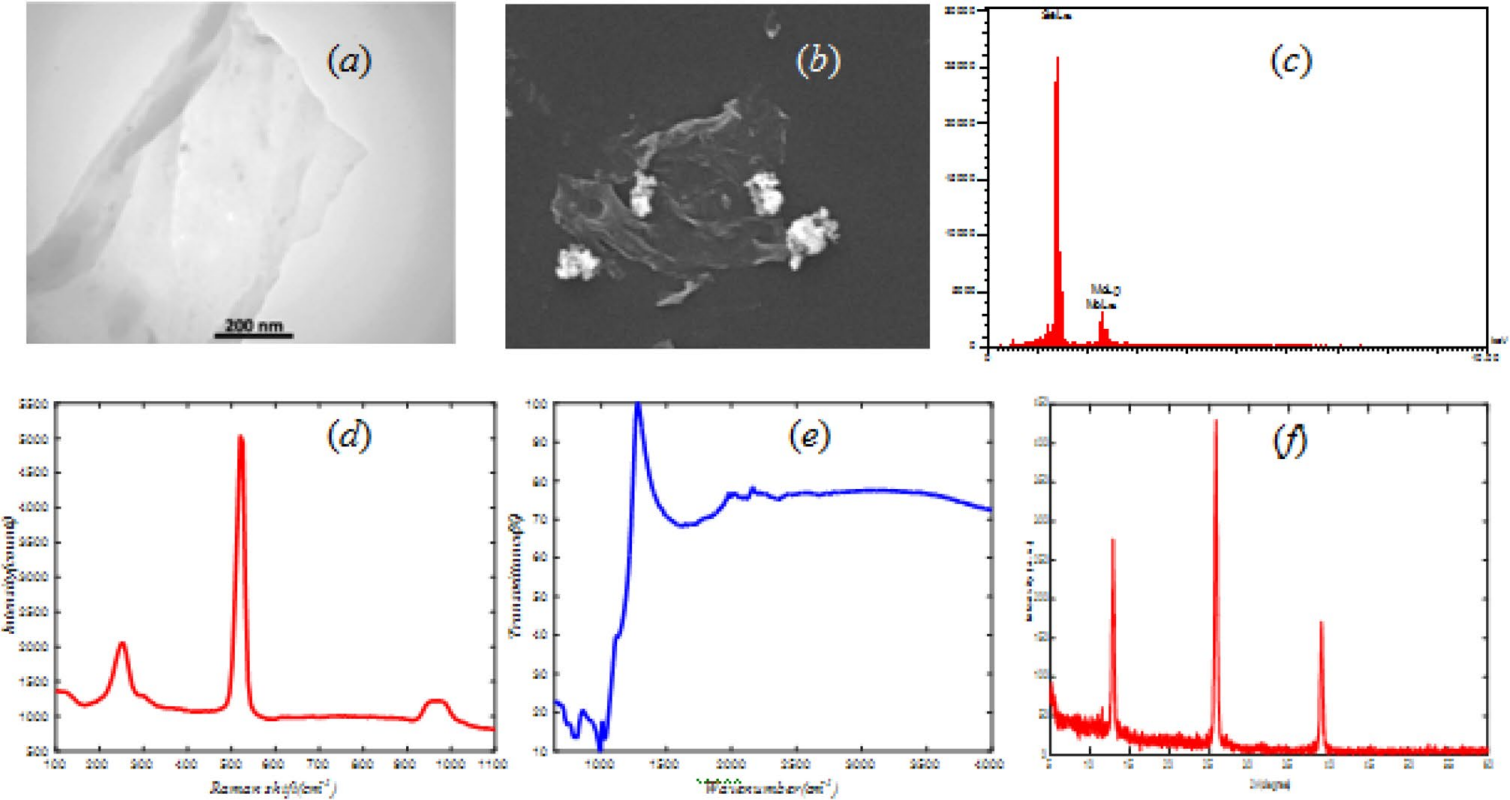
**(a)** TEM, **(b)** SEM, **(c)** EDX, **(d)** Raman, **(e)** FTIR, and **(f)** XRD analysis of MoSe_2_.

Figure 2a,b show the morphology of MoSe_2_ by SEM and TEM images. The few-layer structure of MoSe_2_ is seen in these pictures. Figure 2c implies the presence of Mo and Se in synthesized MoSe_2_ by EDX.

The Raman spectrum in Fig. 2d exhibits two peaks in 240 (cm^−1^) and 290 (cm^−1^) approximately that correspond to off-plane links (A_1g_) and intramolecular links (E_2g_) respectively^31,34^.

Figure 2e shows the graph of FTIR spectroscopy. In this figure 813 (cm^−1^), 992 (cm^−1^) and 1137 (cm^−1^) valleys are related to Mo–O, Mo=O and Se–O bonding respectively^43^.

Figure 2f illustrates the XRD spectrum peaks at 2θ = 13.3°, 25.82°, 39.1°, which can be assigned to the (002), (004), and (103) planes of the hexagonal phase of MoSe_2_, respectively^44,45^.

#### MoS_2_

**Figure 3:**
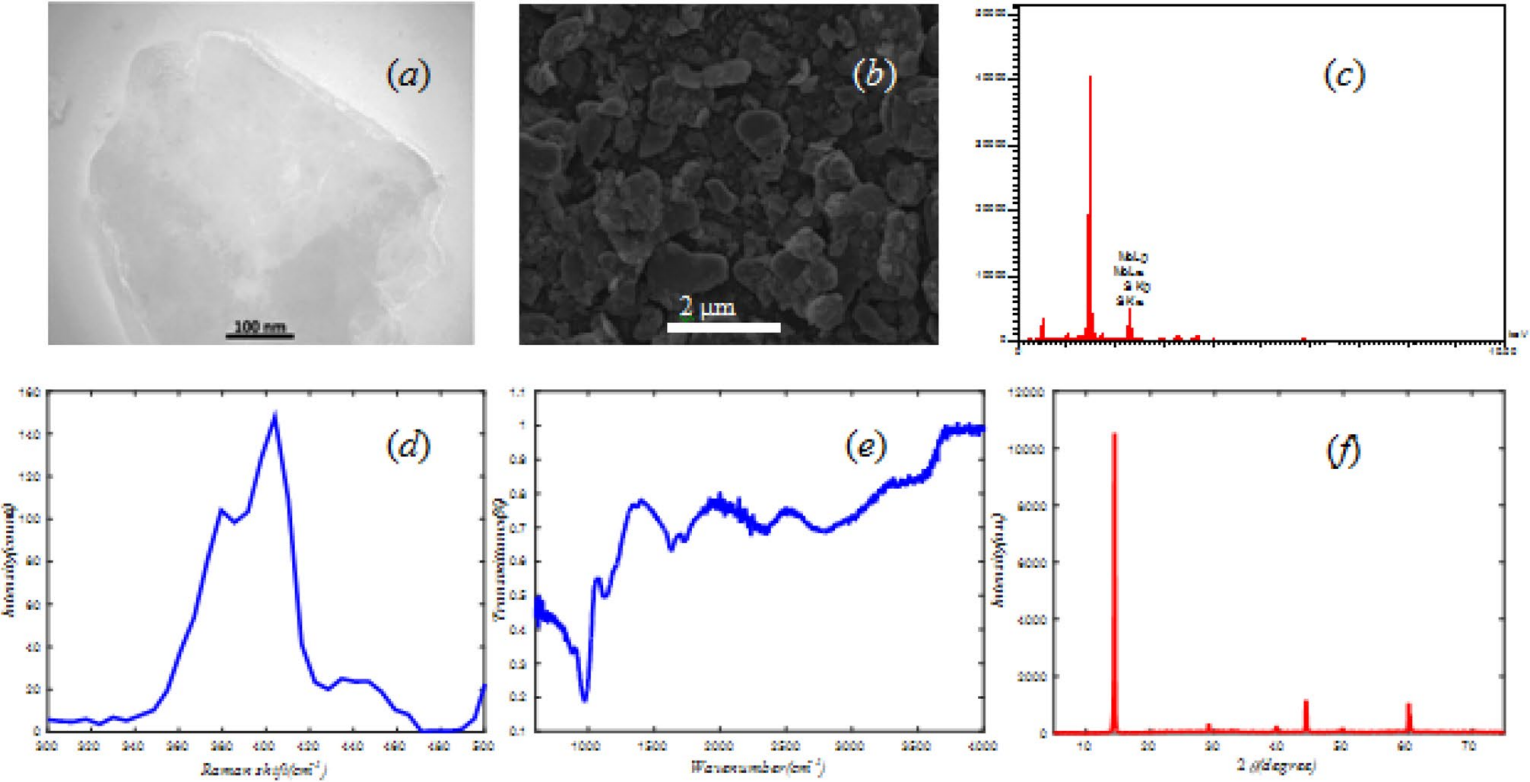
**(a)** TEM, **(b)** SEM, **(c)** EDX, **(d)** Raman, **(e)** FTIR, and **(f)** XRD analysis of MoS_2_.

Two-dimensional MoS_2_ sheets synthesis by chemical exfoliation method. In this method, MoS_2_ was dispersed in a mixture of ethanol and water with 45% and 55% volume ratio. After stirring the composition for 10 min, it was sonicated for 12 h in 40 Hz frequency to separate MoS_2_ sheets from each other. The quality of MoS_2_ layers is investigated by TEM, SEM, EDX, Raman, FTIR, and XRD analysis (Fig. 3).

The SEM and TEM images show the microstructure of synthesized MoS_2_ (Fig. 3a and Fig. 3b) and indicate to broad sheets of MoS_2_. The purity of MoS_2_ sheets is shown in the EDX spectrum (Fig. 3c). The Raman spectra of MoS_2_ layer (Fig. 3d) exhibit two important peaks, the in-plane E_2g_ (~ 381 cm^-1^), and the out-of-plane A_1g_ (~ 405 cm^-1^). E_2g_ mode corresponds to the Mo and S atoms vibrating in one direction, while A_1g_ mode is due to the Sulfur atoms vibrating^14,25^. The difference between these two modes (~ 24 cm^-1^) can be used as a reliable identification for few-layers MoS_2_^46^.

The FTIR spectrum (Fig. 3e) shows the presence of Mo-S, C-H, and Mo–O bands at 600, 1396, and 1639 cm^−1^ respectively. The appearance of a spread peak in the region of 3287 is related to the tensile vibration of OH groups^47^.

The XRD of MoS_2_ sheets in Fig. 3f illustrates the peaks at 2θ = 14◦ (002), 2θ = 32.6◦ (100), 2θ = 39.5◦ (103) and 2θ = 44.2◦ (006), 2θ = 49.8◦ (105), 2θ = 58.3◦ (110). The peaks related to the (002) plans determine the stacking of MoS_2_ single layer by d-spacing of 6.3 nm. Other peaks are characteristic of crystalline nature of MoS_2_ materials^47,48^.

### Fabrication of humidity sensor

**Figure 4:**
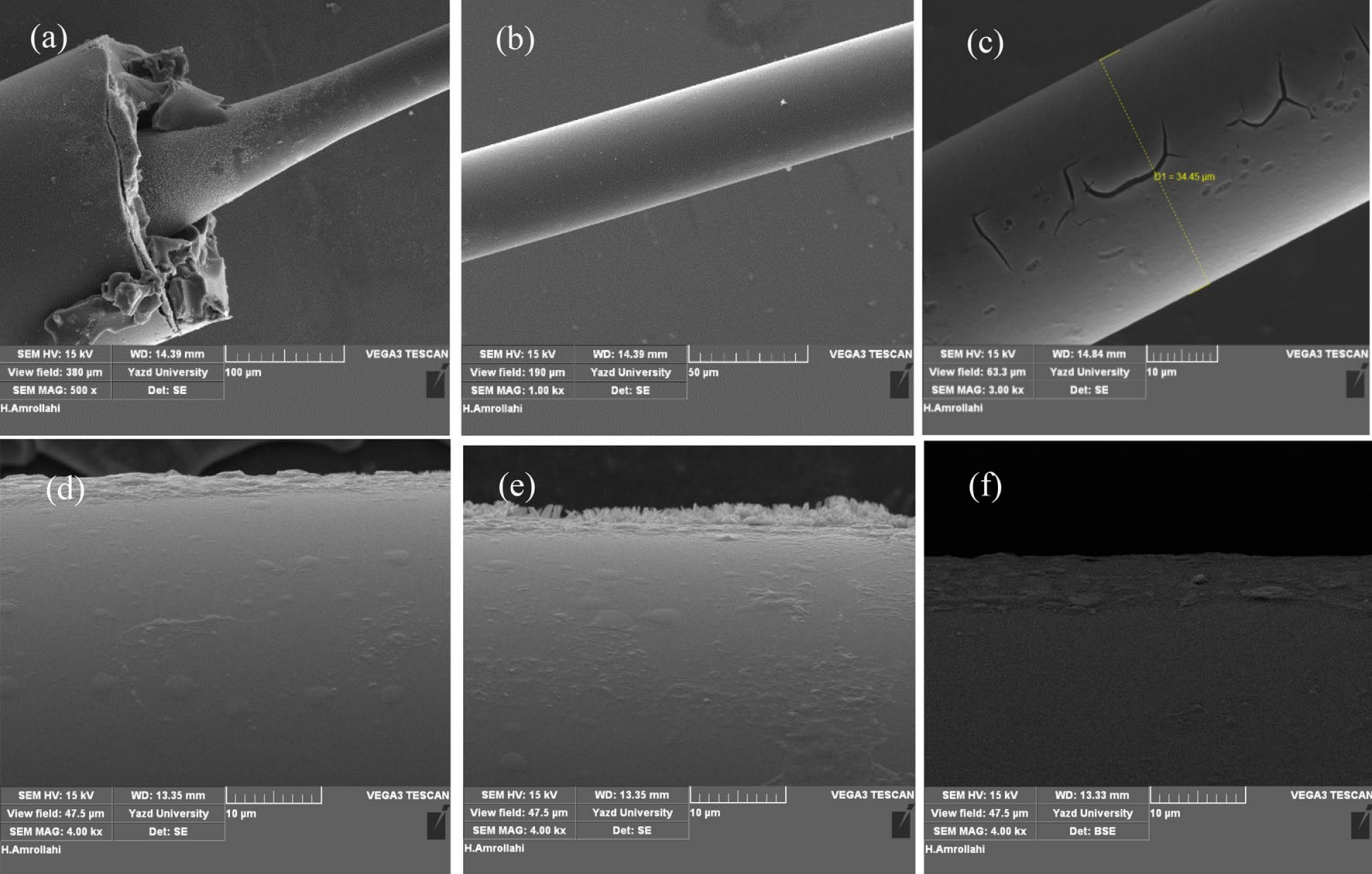
The SEM images of ESMF **(a–c)** and G/GO coated ESMF **(d)**, MoSe_2_ coated ESMF **(e)**, MoS_2_ coated ESMF **(f)**.

In this research, SMF is used as the main part of the optical-humidity sensor. To modify the sensing, the diameter of SMF reduces from 34.45 µm by corrosion. For this purpose, 3 cm of the protective layer of SMF was stripped and wiped up with alcohol, then put it in HF acid for 60 min. The process etched some of the clad-layer on the core of SMF to achieve the desirable evanescence field. Figure 4a–c shows the SEM images of ESMF.

**Figure 5:**
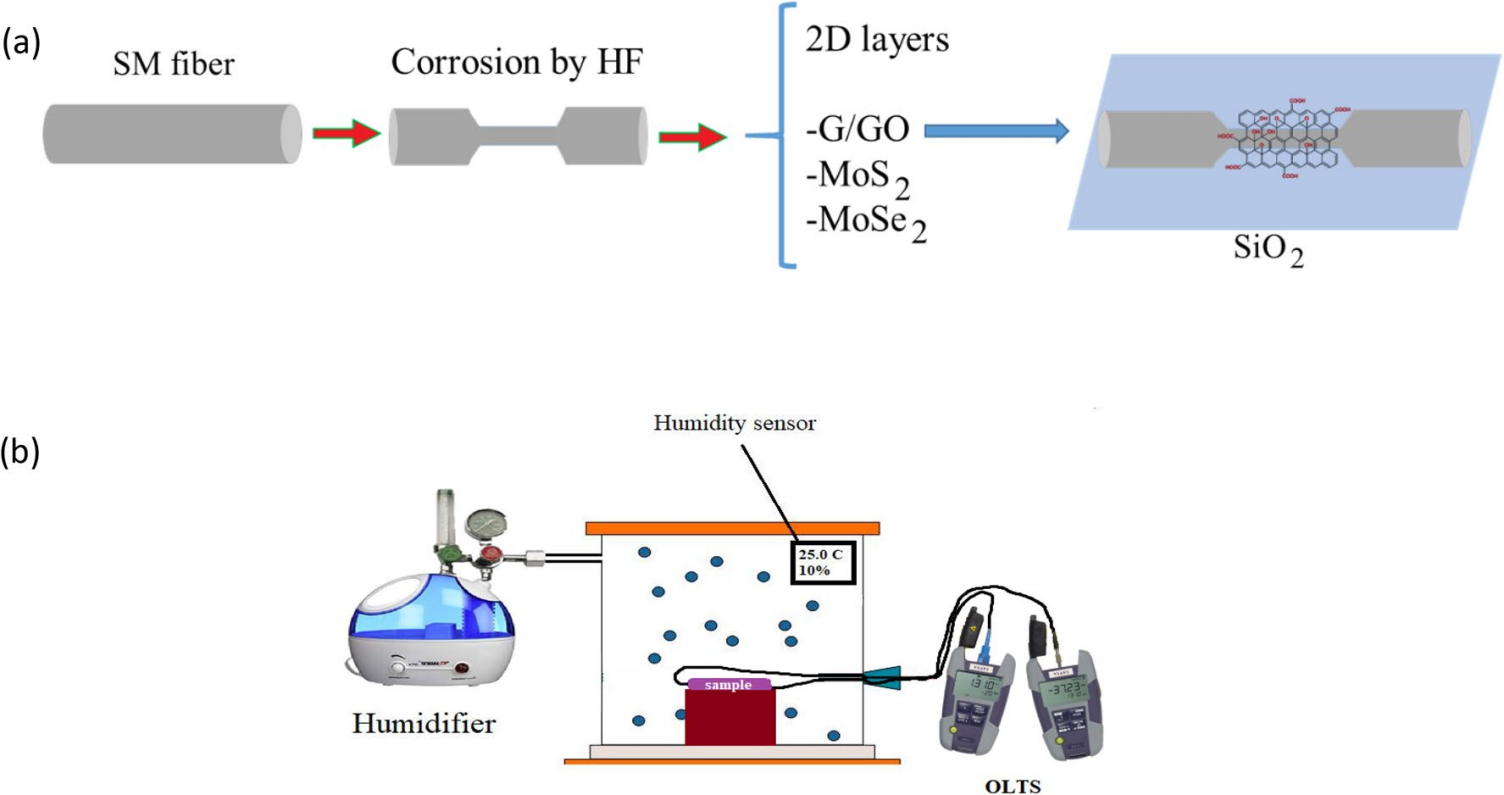
Schematic preparation of **(a)** optical fiber for humidity sensing and **(b)** the setup of the humidity sensing experiment.

In order to make more changes in the refractive index by water absorption, 2D materials have deposited on the surface of ESMF by dip coating in transparent solution contain one or few layers of G/GO, MoS_2_ and MoSe_2_. These solutions are prepared by adding 5 mg of synthesized powders to 10 cc Distilled water separately, sonicating for 2 h, and centrifuging. The SEM images of ESMF coated with G/GO, MoSe_2_ and MoS_2_ illustrate in Fig. 4d–f respectively. Also, the preparation of optical fiber for humidity sensing schematically is shown in Fig. 5.

The OPS and OPM are used as OLTS to analyze RDA, repeatability, sensitivity, and variance versus RH. To create the RH in the range of 20–90% in 25 ∘C, we used a cloud chamber consist of humidifier, humidity control box, temperature controller, and standard humidity sensor as shown in Fig. 5b. Due to the presence of temperature and humidity control system in cloud chamber, these quantities are uniform and constant throughout chamber.

## Result

**Figure 6:**
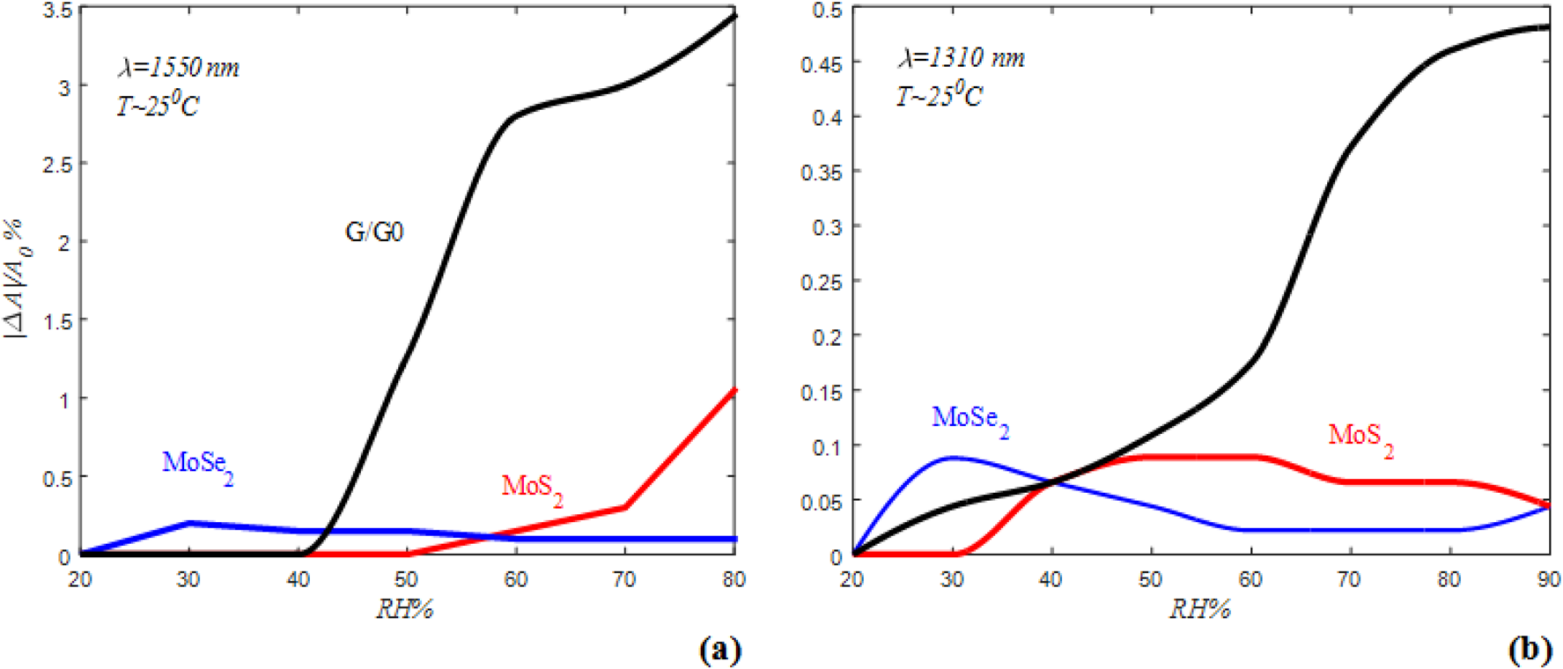
(**a,b**) RDA as a function of RH at room temperature with 1550 and 1310 nm wavelength for different coating respectively.

RDAs as a function of RH for ESMF with different coatings are illustrated in Fig. 6a and b for 1310 nm and 1550 nm wavelengths. As shown in these figures, ESMF coated with MoS_2_ has zero RDA at RH below 30% and at higher RH has lower RDA relative to ESMFs coated with MoSe_2_ and G/GO. So it cannot use as an efficient humidity sensor. Unlike it, the ESMF coated with MoSe_2_ has very good humidity sensing at low RH up to 30%. However, the humidity sensor based on MoSe_2_ is not applicable at medium and high RH due to the lake of the one-to-one function of RH. The ESMF coated with G/GO has high RDA, especially at RH higher than 40%, and also its RDA has the one-to-one function of RH.

The optical humidity sensing mechanism of coated ESMF is related to variation of refractive index by bonding between water molecules and the surface of 2D materials. This physical interaction changes the density of carriers in 2D materials coated on the ESMF and results in the variation of electronic gap energy (E_g_). According to the Penn model, E_g_ are inversely correlated to refractive index^49^.

The presence of functional groups and defects determine the type of synthesized semiconductor powder. For example, impurities such as oxygen, water, carbon dioxide, etc. cause to p-type, and others such as hydroxyl, ether, and epoxide groups convert the intrinsic semiconductor to n-type. So, with respect to the FTIR spectrum (Fig. 1e), G/GO has hydroxyl groups and behaves as an n-type semiconductor. On the other hand, MoSe_2_ has not hydroxyl group and the presence of Mo bonds (Fig. 2e) at the surface makes it a p-type semiconductor. The existence of hydroxyl group and Mo bonds on the surface of MoS_2_ results in the simultaneous presence of acceptor and donor impurities.

The ionization of water molecules occurs as $2H_{2} O \Leftrightarrow H_{3} O^{ + } + OH^{ - }$ near the surface by polar bonds. The negative part produces the water molecules by interacting with the Hydrogen in the air ($2OH^{ - } \,\, + \,H_{2} \,\, \to \,2H_{2} O$). The positive part of it interacts with the hydroxyl group on the surfaces and excites as water molecules ($H_{3} O^{ + } \,\, + \,OH^{ - } \,\, \to \,2H_{2} O$). So, the adsorption of water molecules on the surface changes the carrier densities of it by withdrawing electrons.

Taking out the electrons from a p-type surface like MoSe_2_, by water adsorption, decreases the refractive index because of the increase in E_g_. This mechanism in G/GO as an n-type semiconductor is vice versa. However, MoSe_2_ coated ESMF at low humidity adsorb H_2_O and show high RDA relatively, and saturate in RH about 30%. At higher humidity, MoSe_2_ saturate and will not adsorb more H_2_O. As regards, the functional groups at G/GO can absorb H_2_O up to 90% RH and the its refractive index increase by adsorption of water molecules on G/GO coated ESMF because of the increment of carrier densities.

The MoS_2_ coated ESMF has low RDA and indeterminate humidity sensing behavior because of competition between donor and acceptor of electrons impurities on its surface.

As shown in Fig. 6**,** RDA of G/GO in the telecommunication’s standard wavelengths, 1310 nm and 1550 nm, is higher than MoSe_2_, and MoS_2_. In addition to the changes in the density of carriers with respect to changes in relative humidity, the adsorption of water molecules between the graphene oxide sheets cause to change in refractive index and improve its humidity sensing^50^. So other quantities just measure for G/GO-based ESMF.

**Figure 7:**
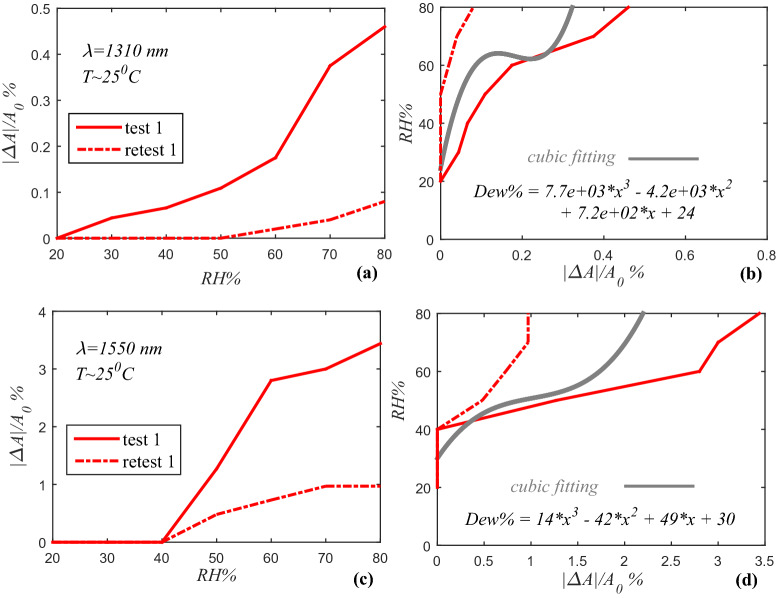
Repeatability of RDA as a function of RH for G/GO in 1310 nm (**a**) and 1550 nm (**c**) wavelengths; reversing and fitting curves to calculate RH formulation in 1310 nm (**b**) and 1550 nm (**d**) (dash-line curves are rapid retest of line curves).

The repeatability curve shows a reduction of RDA during consecutive tests (Fig. 7a,c), due to saturation hydrogen bonding in G/GO structures. It seems thermal treatment of G/GO coated ESMF at 90 °C can reactive the sensor because of the separate the oxygen and water molecules from the G/GO surface^16,51^. To calculate the formulation for measuring RH from Attenuation, reversing curves are illustrated and fitted by MATLAB, which are shown in Fig. 7b,d.

**Figure 8:**
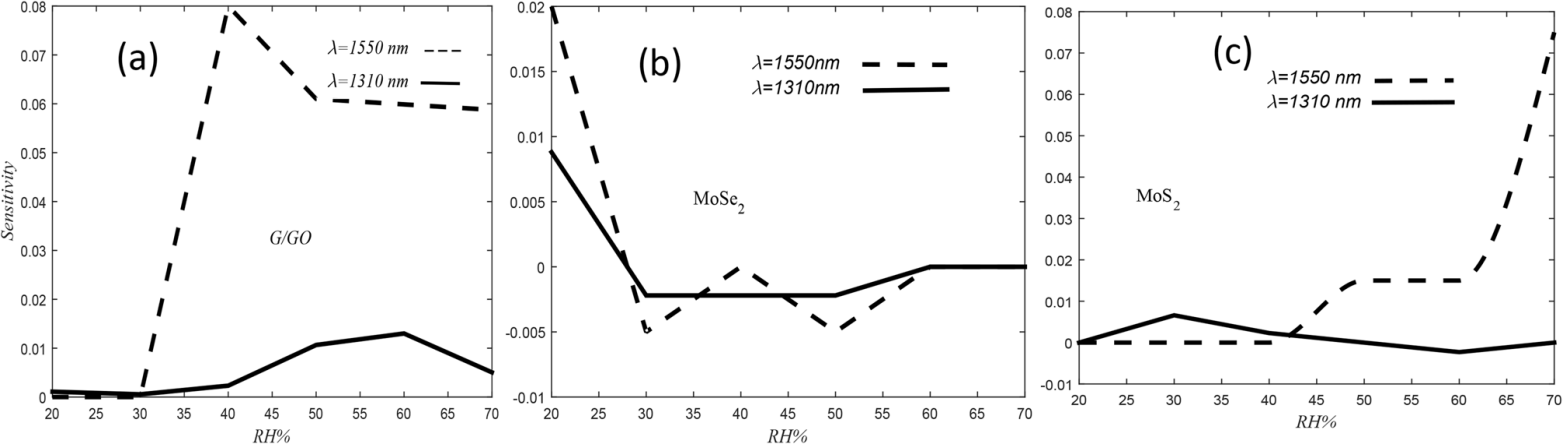
Sensitivity of humidity sensors coated with **(a)** G/GO, **(b)** MoSe_2_ and **(c)** MoS_2_ in both 1310 nm (line curves) and 1550 nm (dash-line curves) wavelengths.

Sensitivity has defined as a ratio between the differential of the output signal and measured properties (humidity)^52^. Sensitivity for the humidity sensors coated with G/GO, MoSe_2_ and MoS_2_ are illustrated in Fig. 8. It is obvious from this figure, sensitivity of G/GO (MoS_2_) coted ESMF at RH more than 30% (60%) increases and has more magnitude in 1550 nm wavelength relative to 1330 nm wavelength. While, MoSe_2_ coated ESMF has very low sensitivity at both wavelengths.

**Figure 9:**
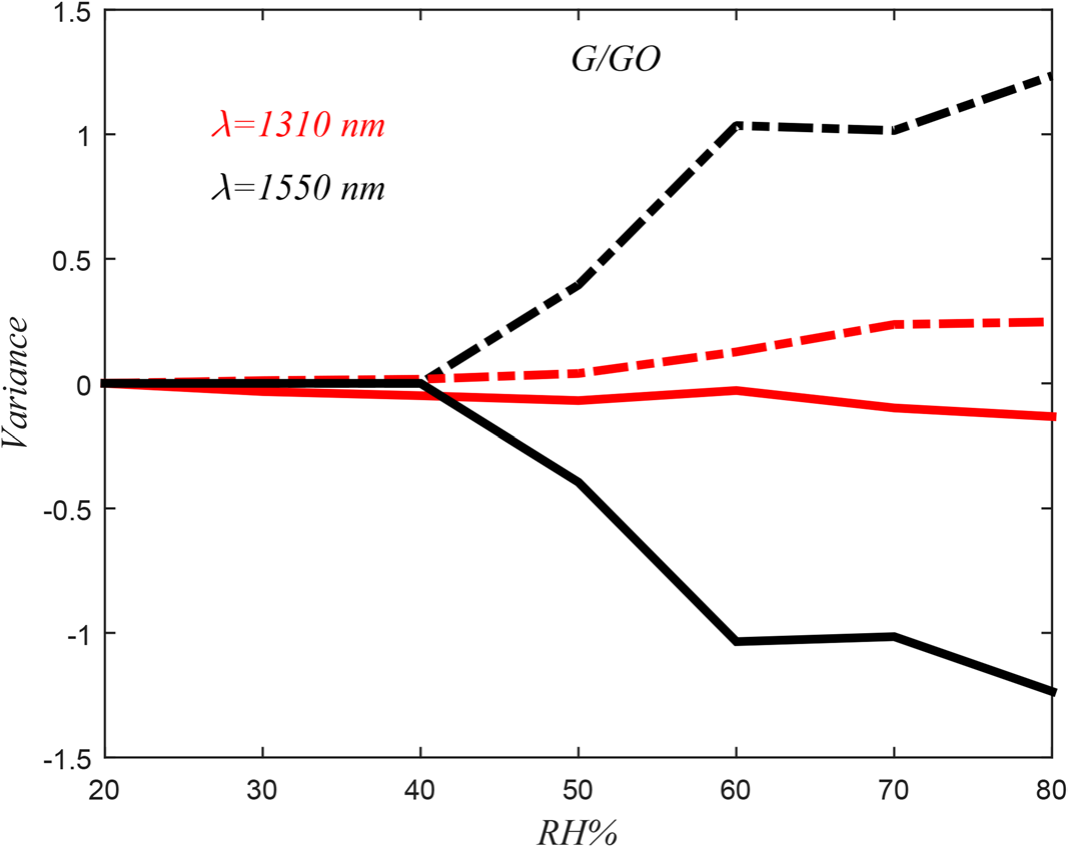
Variance as a function of RH from average values of G/GO-based sensor in 1310 nm and 1550 nm wavelengths.

Variances of any test from the average value are plotted in Fig. 9. As shown**,** variance for the investigated sensor is small in the RHs less than 40%. In addition, variance is so little in 1310 nm wavelength than 1550 nm. So, results obtained from the humidity sensor based on etched-fiber coated with G/GO have better accuracy at low humidity and 1310 nm wavelength.

## Conclusions

In this research, ESMF that coated by G/GO, MoSe_2_, and MoS_2_ to application as humidity sensing are prepared. The RDA, repeatability, sensitivity, and variance analyzes are investigated versus RH at both standards wavelengths of telecommunication (1310 nm and 1550 nm). Results show that the sample coated with MoSe_2_ has higher RDA at low humidity (less than 30%). So it can be used as a low humidity sensor. However, the sensor based on MoSe_2_ is not practical at humidity more than 30% due to low and lake of the one-to-one function of RDA versus RH. The ESMF coated by MoS_2_ has indistinctive behavior by variation of RH because of n-type and p-type impurities on the surface of MoS_2._ The best humidity sensor, in this research, is the ESMF that coated by G/GO. It has partially high and one by one function RDA versus RH. The sensitivity for it is reasonable and the low variance shows its accuracy. Also, repeatability can modify by thermal treatment.
